# The PFAS Conundrum—Of Logic, Science, Policy

**DOI:** 10.3390/toxics13100885

**Published:** 2025-10-16

**Authors:** Jaap C. Hanekamp, Lucas Bergkamp, William M. Briggs, Moira Wilson

**Affiliations:** 1 Independent Researcher, 2725 NA Zoetermeer, The Netherlands; 2 Independent Researcher, 1000 Brussels, Belgium; lucasbergkamphw@gmail.com; 3 Independent Researcher, Charlevoix, MI 49700, USA; matt@wmbriggs.com; 4 Certified Group, 3436 ZZ Nieuwegein, The Netherlands

**Keywords:** PFAS, REACH, chemical ban, restrictions of hazardous substances, toxicology, chemistry

## Abstract

The comprehensive (“universal”) PFAS ban proposed by the EU raises questions of a scientific, philosophical, regulatory, and policy nature. This overview scrutinizes the proposal and finds it wanting in each of these respects. The grouping of all PFAS is based on a methodology that is only loosely science based. The scientific data on PFAS do not support the open-ended definition and the drastic restrictions that would be imposed by the proposal. To illustrate the weak scientific basis, we look more closely at the immunotoxicity claims as found in a few landmark epidemiological papers. We find these claims not well-founded and methodologically lacking. Also, we scrutinize a few animal studies and comment on their results. Detecting PFAS in multiple matrices is briefly looked at. The analytical context of detectability, quantifiability, measurement precision, and reproducibility of results of PFAS present in especially complex matrices (e.g., foods, soil, waxes, and fats) poses quite the challenge. Experimental uncertainties are reported to be extremely high. Disregarding the key distinctions between hazard and risk and between potential hazard and hazard, the proposal treats all PFASs, broadly defined, as presenting hazards based on an assumed common property: persistence. On this and other grounds, including the requirement of “unacceptable risk”, the proposed ban fails to meet the requirements imposed by the REACH Regulation. From a public policy perspective, the costs of the proposal would appear to eclipse any advantages for human health and the environment. Thus, we conclude that the proposed PFAS ban is problematic from these key perspectives.

## 1. Introduction

In February 2023, the European Chemicals Agency (ECHA) published a proposed ban on per- and polyfluoroalkyl substances (PFASs). This proposal is built around emerging evidence on the hazards posed by these chemicals to the environment and human health. This evidence forms the centerpiece of our discussion on the hazards of PFASs. Before we come to that, we will first have some introductory notes on the topic.

PFASs are diverse classes of thousands of synthetic chemicals that include gases, liquids, and solid high-molecular-weight polymers. They are used in many applications and products, varying from automotive and aviation to food contact materials, textiles, construction and household products, electronics, firefighting foams, and medical articles. PFASs have unique desirable properties, such as excellent stability under intense heat, and are simultaneously water and grease repellents. We first expound the basis for this extensive regulatory attempt pursuant to the EU’s REACH Regulation. Inspired by the precautionary principle, the REACH regime is increasingly being conceived as the way to a “toxic-free” environment or, to the extent that this is unachievable, at least to a society which maximally reduces the hazards arising from synthetic chemicals [1]. In the EU’s Green Deal, this ideal is reiterated as follows.

“Chemicals are everywhere in our daily life. They form part of nearly all device we use to ensure our well-being and protect our health. Chemicals are the building blocks of the low-carbon, zero pollution, energy- and resource-efficient technologies, materials and products that we need for making our society and economy more sustainable. At the same time, chemicals can have hazardous properties that harm human health and the environment. They can cause cancer, affect the immune, respiratory, endocrine, reproductive and/or cardiovascular systems, weaken human resilience and capacity to respond to vaccines and increase vulnerability to diseases” [2].

The Chemicals Strategy for Sustainability sets out the steps to achieve a toxic-free environment, and ensures that chemicals are produced and used in a way that augments their contribution to society while avoiding harm to the planet and to current and future generations [3]. The Strategy contemplates that the most harmful chemicals are avoided for non-essential societal use, and that all industrial chemicals are used safely and sustainably. In parallel, the strategy is intended to promote the green transition of the chemical sector and its value chain. A toxic-free environment is also part of the Commission’s Zero Pollution Ambition for air, water, and soil [4].

The EU’s ambition raises the question of how a toxic-free environment is to be understood logically. Chemically and toxicologically, “toxic-freeness” as such does not seem to be an articulate concept, since any substance can have both desirable and undesirable effects, depending on use, dose, exposure routes (e.g., oral dermal), dose-time, exposed organisms and organs, bioavailability, and other relevant parameters. Indubitably, preventing industrial chemicals from ending up (un)intentionally in the environment is quite a desirable objective embedded in many regulatory structures. Now, the practicality of restricting a chemical with known undesirable effects on human and environmental health that outrank any known (or projected) industrial and societal benefits at relevant environmental concentrations will not be discussed here.

If, however, industrial pollution needs to be curbed beyond known thresholds of toxicity, that is, beyond the Paracelsian paradigm, it would call for a precautionary intervention of the detectable presence of industrial chemicals in the environment and human beings. As limits of detection have plummeted to the levels of even ppq—parts per quadrillion, measurements in picograms per kg—new and still unanswered questions arise as to the toxicological meaning and regulatory latitude of such concentrations. Nevertheless, the projected precautionary ban on most PFASs is in principle viewed as both an achievable and a desirable ideal. Any such goal cannot be treated as absolute, however, since such a precautionary approach will impose intended and unintended costs and risks, thus creating risk/risk trade-offs by default [5]. Additionally, the Second Law of Thermodynamics (the progression towards thermodynamic equilibrium) drives the inexorable diffusion of all chemicals throughout the world, irrespective of source.

First, however, we will characterize PFASs, and subsequently, we will concisely assess the toxicology of PFASs that is the most relevant to the proposed ban. That requires at least a closer look at key animal and epidemiological studies that have served as markers in the PFAS discourse. The legal and regulatory implications of the proposed ban will be addressed as well.

## 2. Per- and Polyfluorinated Substances—Regarding Chemistry, Physics, Degradability, and Analytics

PFASs, a chemical group of substances defined primarily by carbon and fluorine, contain thousands of diverse synthetic chemical substances with a variety of uses [6]. These chemicals can be gases, liquids, and/or solid polymers. PFASs are hugely varied and are used in a wide range of applications, including but not limited to firefighting foams, electronics, textiles (a variety of clothing, carpets, furniture, etc.), and construction, in industries like surfactants and household products, such as cookware, as well as food packaging and cosmetics. PFASs are valuable for their unique ability to repel both water and oils, as well as being stable in high-temperature environments. Two large subcategories are long-chain and short-chain PFASs, even though this is merely a crude taxonomy. Long-chain PFASs are generally categorized as having eight or more carbon atoms, while short-chain PFASs contain fewer than eight.

PFASs, as chemically defined by the OECD [7], are specified as fluorinated substances that contain at least one fully fluorinated methyl or methylene carbon atom (without any H/Cl/Br/I atoms attached to it). In other words, with a few noted exceptions [7], any chemical with at least a perfluorinated methyl group (–CF_3_) or a perfluorinated methylene group (–CF_2_–) is a PFAS. PFASs are chemically distinct by their very strong carbon–fluorine covalent bonds. The high electronegativity of fluorine gives the carbon–fluorine bonds a substantial dipole moment. This introduces, apart from the covalent nature, ionic character to the bond through partial charges (C^δ+^—F^δ−^). The partial charges on the fluorine (δ−) and carbon (δ+) are attractive, contributing to the unusual bond C-F strength.

Per- and polyfluoroalkyl substances differ slightly in their structure. In perfluoroalkyl substances, all carbon atoms except one are bonded to fluorine atoms, in contrast with polyfluoroalkyl substances, where at least one, but not all, carbon atoms are bonded to fluorine atoms. Fluorine, despite being the most abundant of the halogens in the earth’s crust, is only found sparingly in natural chemicals, although other organohalogens with Cl and Br are found ubiquitously as natural products [8]. Exceptions are, e.g., fluoroacetic acid and w-fluorinated fatty acids [9].

Resistance to environmental degradation is largely due to the unusually strong C-F bond, making microbial biodegradation difficult. Collivignarelli et al., for instance, investigated the inhibitory effects of PFASs (PFOA; PFOS) on activated sludge biomass at a short-term exposure (10 min contact time in batch tests) and medium-term exposure (several hours in continuous tests) [10]. The high concentration range—between some 50 mg/L and 200 mg/L—was chosen by the authors based on values representative of heavily contaminated (industrial) effluents. The authors reported that waste containing PFAS did not exert inhibitory effects on the aerobic biomass, suggesting the inertness of PFASs. Zhang et al. reviewed the biodegradation of a variety of PFASs [11]. They show a range of degradation efficiencies depending on microorganisms, test duration, and matrix conditions (aerobic and anaerobic). The authors conclude that both bacterial and fungal species are effective in the degradation of PFAS species, and that biodegradation of PFASs can occur with or without C-F bond cleavage. In line therewith, the European Food Safety Authority remarks that “among the various PFASs, some groups of compounds are extremely persistent such as PFCAs and PFSAs while others are easily degraded in the environment and in humans” [12]. In summary, there is variability in PFAS persistency, and classification should be in line with this very same variability.

The detectability, quantifiability, measurement precision, and reproducibility of results of PFASs present in especially complex matrices (e.g., foods, soil, waxes, and fats) pose quite the challenges and seem to lack academic discussion as to further this field of research. The Nordic Council of Ministers, for instance, reports that available analysis methods of PFAS partly have a limit of quantification (LOQ) above the legal restriction limit value, which in and of itself is quite problematic [13]. Also, experimental uncertainties are reported to be extremely high. This is especially true for challenging matrices of various kinds [13]. This means that determining toxicity thresholds for multiple relevant endpoints is challenging. Furthermore, Idowu et al. (2025) report that limitations of total PFAS methods include contradictory results when complementary techniques are applied to the same sample, potentially leading to over- or underestimation [14]. To be clear, the quality and reproducibility of analytical results, combined with estimated measurement uncertainties, are central in discussions on the (eco)toxicity of PFAS concentrations found in humans and the environment. These findings, at least, complicate the ascertainability of enforceable toxicity standards. That brings us to the European Chemicals Agency’s (ECHA) Annex XV Risk Inventory [15].

## 3. The Annex XV Risk Inventory—Reasoning and Methodology

“The outstanding property of all PFAS is their extraordinary persistence” [16]. This property is possibly the biggest toxicological concern regarding PFASs, both with respect to environmental and human hazards. That being said, persistency and mobility [17], per se, do not equate with hazard or risk. More precisely, persistency is not a toxicological claim. It is a physico-chemical claim. In fact, persistency is an expedient feature for many physiological processes, such as oxygen transport, wherein the element iron (in its 2+ valency) functions as an enduring chemical platform. Nevertheless, in the case of synthetic (but not naturally occurring) chemicals (and elements), persistency is typically equated with inherent or at least potential harm to the environment or humans. For a persistent chemical to be hazardous, the bioaccumulation potential ánd the relevant (eco)toxicity must be considered jointly [7]. If toxicity at relevant concentrations does come into play, persistency will indeed be a major cause for concern.

This brings us to the key challenge for a universal PFAS ban, which is to justify the scope of the proposal. What line of argument could be put forward to classify all PFASs posing an “unacceptable risk” while there is data only for a fraction of these substances? The dossier submitters put forth the following, to which we will add some preliminary comments:

— Persistence is a common property of all PFASs as they share the same fundamental physico-chemical characteristic: a strong carbon–fluorine bond, which makes them resistant to degradation. As we showed earlier, persistency varies widely across the PFAS spectrum, which begs the question against the overarching persistency criterion.
— Said persistency implies that once any PFAS is released into the environment, it will not break down and subsequently will accumulate over time, leading to increasing and irreversible contamination of water, soil, and air. The unresolved question here is whether the presumed logic of this causal chain of events should trump (the lack of) empirical data for most PFASs.
— Persistence and subsequent potential accumulation together constitute environmental and human health risks that need to be addressed. Persistency, per se, again, does not constitute a hazard. For some hazard to be identified at all, data on bioaccumulation and toxic effects must be presented. The proposition that accumulating PFASs will eventually cross some no-effect level will require data on bioaccumulation and toxic effects, which are lacking for most PFASs.

The dossier submitters further argue that, given the irreversible nature of contamination and the potential for long-term, still-unknown harm, waiting for substance-specific data on all PFASs would take too long and would cause the environmental and health burden to become unmanageable. As a stopgap for lack of data, proponents invoke the precautionary principle as a means to avoid regrettable substitution. That is, if only some PFASs are restricted, industries will likely switch to other PFASs that may be just as persistent and possibly just as harmful. Again, despite its apparent merit, it seems that this argument begs the question. Any substitution might be regrettable, and precaution, per se, will not guide us towards a safer chemical replacement. In fact, precaution provides no guidance at all. Sunstein famously remarks the following:

“The real problem with the Precautionary Principle … is that it is incoherent; it purports to give guidance, but it fails to do so, because it condemns the very steps that it requires. The regulation that the principle requires always gives rise to risks of its own—and hence the principle bans what it simultaneously mandates. I therefore aim to challenge the Precautionary Principle not because it leads in bad directions, but because read for all it is worth, it leads in no direction at all. The principle threatens to be paralyzing, forbidding regulation, inaction, and every step in between. It provides help only if we blind ourselves to many aspects of risk-related situations and focus on a narrow subset of what is at stake. That kind of self-blinding is what makes the principle seem to give guidance; …” [18].

Overall, the PFAS ban, for a substantial part, relies on three worst-case proxy arguments: as some PFASs are evidently persistent, then all must be, in view of their basic chemical structure; PFAS persistency for some is a bio-accumulative hazard for all; banning only some known hazardous PFASs might induce regrettable substitution with other and newly developed PFASs.

## 4. Immunotoxicity of PFAS—Some Critical Notes

All the above makes it even more important to review at least a subset of human health data for PFAS that has specifically caught the attention of regulators and scientists alike. The Annex XV Restriction Report of 22 of March 2023 proposes the reduced vaccine responses in children as the most important health impact in immune outcomes [19]. As immunity against a range of diseases is an overarching personal and societal concern, we will concisely review this topic. We will take this assessment by ECHA as a given and the 2020-EFSA review “Risk to human health related to the presence of perfluoroalkyl substances in food” as a point of departure [12]. This substantial EFSA report notes four endpoints as “potential critical effects for PFOS and/or PFOA”. Effects on the immune system, as shown by decreased antibody responses to vaccines, is one of those four. The EFSA Panel concluded that “effects on the immune system, which were observed at the lowest serum PFAS levels in both animals and humans, is the critical effect. The findings were considered robust since in animals they were consistently observed for two studied PFASs (PFOA, PFOS) and also in humans” [12].

In animal studies, Qazi et al. (2009 a, b), for instance, found several changes in immune parameters at circa 40 mg PFOS/PFOA per kg body weight per day for 10 days (C57BL/6 mice) [20,21]. Vetvicka and Vetvickova found effects on the immune system after short-term exposure to PFOS and PFOA at 20 mg/kg body weight per day for 7 days [22]. Guruge et al., prior to inoculation with an influenza virus, administered PFOS to B6C3F1 mice for 21 days at 5 and 25 µg/kg body weight per day [23]. Survival rates between day 14 and day 20 after virus infection were dose-dependently reduced: some 18% for the highest dose and some 46% for the controls. At least, from these four studies as referred to in the EFSA report [12], PFASs such as PFOS and PFOA do influence the immune system of test animals, albeit at doses that are typically well outside the ranges of human exposures. To illustrate the latter, Göckener et al. report PFAS concentrations in human plasma to be between 0.27 and 14.0 ng/mL for PFOA (2009–2019) and 1.21–14.1 ng/mL for PFOS (same time interval) [24]. No measurement uncertainties were reported by the authors.

Another line of evidence put forward in the PFAS immunotoxicity discourse comes from epidemiological data. Below, we will tackle some epidemiological papers put forward by EFSA [12]. The Grandjean et al. paper examined changes in antibody concentrations of two vaccinations, diphtheria and tetanus, in approximately 500 children at 7 and 13 years of age [25]. It is expected that, on average, due to natural causes [26,27], irrespective of PFAS exposure, these concentrations would naturally decrease, although they may increase in some children. And they would increase even more if children had boosters. It is surmised by Grandjean et al. that “PFAS exposure” causes a decrease in antibody concentrations from 7 to 13 years. If that assumption is accurate, any observed decrease (or change in any direction) may be due to natural causes, to PFAS exposure, or to both. No attempt was made by the authors to separate a potential PFAS effect from natural causes. PFAS concentrations were also measured at both points. The paper reports that 68 children had ER visits during this period, and the authors supposed these children had booster shots, but that assumption was not verified. Plots of antibody concentrations at 7 years against antibody concentrations at 13 years were shown for all children.

The authors do not show plots of PFAS and antibody concentrations or changes in concentrations, and no relevant data are added in the supplementary files. Such plots, which are easy to produce (and are like the ones they did include), would go a long way toward corroborating their hypothesis of a potential causal link between PFAS exposure and decreased antibodies. In the analysis presented, however, readers are asked to rely solely on model output to arrive at their judgment. Model 1 is a regression of anti-diphtheria, with the “change in antibody concentration as expressed in percent per doubling of the serum PFAS concentration at the two different ages”. The paper shows no important signal with any PFAS or change in antibody concentrations. To “improve” upon their hypothesis, the authors removed the children who went to the ER and were suspected of having had boosters. The analysis scarcely changed: no important signal with any PFAS and no change in antibody concentrations was found. Then, in a curious move without any reported justification, the authors excluded both children who visited the ER and those children (202 in total!) who had increases in antibody concentrations, which had the effect of biasing the sample to (mostly) those kids who went down in antibody concentrations. The analysis was repeated, and a small signal was realized in one PFAS—PFDA.

Model 2 is a regression of an anti-tetanus “change in the antibody concentration as expressed in percent per doubling of the serum PFAS concentration at the two different ages”. The corrected result is the same as for anti-diphtheria: a null finding at 13 years old for all PFASs, and a null result for all PFAS for 7-year-olds, except for PFDA. It therefore may seem there is something to the PFDA signal since it was also found in anti-diphtheria. However, it is only found in the complete sample, and not in the ER-reduced sample, and not in the fully reduced (no ER, no decreases) sample, where we would expect it, because these reduced samples are (hypothetically) prone to having decreases in antibodies.

The Abraham et al. paper fares no better [28]. No data is presented in or with the paper; only model outputs are given. Again, no plots of serum PFAS levels and actual antibody levels, or the actual changes in antibody levels, are presented. A “significant” regression is insufficient, especially when the analysis uses a technique that is not reproducible (i.e., the stepwise approach, which is notorious for over-fitting). We are not told what went into the model. When considering real-world immunity issues—disease—the authors remark that “no associations were observed between levels of PFOA/PFOS measured at the age of 1 year (postnatal exposure) and the number of infections within the first year of life. This may be due to levels of PFASs being not high enough to cause this effect or due to a protective influence of the long duration of breastfeeding in the higher exposed children, if indeed an impact on the occurrence of infections is to be expected at higher levels of exposure”. Corroboration for this proposal is, however, not given.

The paper by Fei et al. analyzed the associations between maternal concentrations of PFOS and PFOA and risk of hospitalization due to infectious diseases [29]. EFSA remarks that “no associations were observed between maternal concentrations of PFOS and PFOA and offspring hospitalisation” [12]. However, the authors themselves report that the “relative risks of hospitalizations ranged from 0.71 to 0.84 for the three higher quartiles of maternal PFOA levels compared with the lowest”. That would mean, if taken at statistical face value, that PFASs would be protective against hospitalization for infectious diseases. Goudarzi et al. looked at prenatal exposure to perfluoroalkyl acids (PFAAs) and the prevalence of infectious diseases in children up to 4 years of age [30]. EFSA reports that Goudarzi et al.’s study, amongst others, “found some positive associations between maternal concentrations of PFASs with offspring’s later risk of infections” [12]. In the authors’ Figure 1, only a few of the Odds Ratios might be “significant” (not crossing 1) after separating boys and girls [30]. Further, PFAS numbers were analyzed per quartiles, and then checked for ORs inside each quartile (compared against the first quartile). Despite that, the signals were quite low. Further modeling delivered only a few small *p*-values. Lastly, Impinen et al. associated prenatal exposure to perfluoralkyl substances (PFASs) with respiratory tract infections [31]. The authors determined 17 disease outcomes (“different allergy- and asthma-related endpoints”) with six different PFAS on which they performed 17 regressions (see their Table 4). Only five small *p*-values were reported. Just as with Goudarzi et al., the authors should have corrected for multiple testing (e.g., Bonferroni), but did not.

A recent paper by Antoniou and Dekant concluded that epidemiologic evidence does not suggest a “strong association between PFAS and immune conditions or infectious diseases in humans. Some associations were observed, but they lacked consistency across studies” [32]. A year earlier, Garvey et al. concluded that “while there is some evidence that PFOA and PFOS may be immunotoxicants, the current human data are not sufficient to support the use of human data in the derivation of reference dose values, and that the animal data suffer from limitations including the predominant use of high dose, short-term exposures in a single strain of mouse with uncertain human relevance/concordance” [33]. Garvey et al. also note that “data from the Faroe Islands cohorts [25] should not be used as the primary basis for deriving PFOA/PFOS toxicity values for human health risk assessments”.

## 5. The PFAS Ban in Regulatory Perspective—Overlooking REACH?

The proposed PFAS ban raises issues from a regulatory perspective. Because the ban would be a restriction under Article 68 of the REACH Regulation, it would have to be demonstrated that PFASs as a group cause an “unacceptable risk to human health or the environment that is not adequately controlled”. In addition, this unacceptable risk must arise from “the manufacture, use or placing on the market of substances” and it “needs to be addressed on a Community-wide basis” [15]. The term “unacceptable risk” is not defined in the REACH Regulation [34], but it could be interpreted as the flip side of “adequate control” [15]. Risk is not defined either, but it is clear from the structure of the REACH Regulation that risk means hazard plus exposure. ECHA takes the position that the term “unacceptable risk” covers not only the situation where the risks of a specific substance are not adequately controlled, but also cases in which the risks of each of several individual substances are adequately controlled, but the aggregated exposure would create an unacceptable risk [15]. PFASs would fall in the latter category—the combined effects of multiple PFASs would pose unacceptable risk to health and the environment.

The PFAS proposal, however, does not validate that releases of PFASs jointly create unacceptable risk. Rather, the proponents posit that “due to the non-threshold nature of the hazards, the risks cannot be quantified, and current releases of PFASs should be minimised”. As the release of PFASs should be considered a proxy for risk, due to the ongoing releases, the risks associated with PFASs would currently not be adequately controlled. Based on an assumption of “unacceptable risk for human health and/or the environment identified for the group of PFASs, measures are needed to minimize the releases to the environment and reduce human exposure to a minimum” [19]. The proponents support their argument with the assertion that “PFASs should be treated as non-threshold substances for the purpose of risk assessment, similar to PBT/vPvB substances under the REACH Regulation, with any release to the environment and environmental monitoring data regarded as a proxy for an unacceptable risk”. Since the proposed restriction “enables a regulatory path to prevent the increase of general PFAS exposures” [19], it would meet the legal condition of “unacceptable risk”. Preventing exposures to substances, however, is not the same as addressing unacceptable risk.

The legal standard of “unacceptable risk” requires more than a possibilistic argument. Even where the precautionary principle is invoked, policymakers must ensure that “as thorough a scientific evaluation of the risks as possible” is conducted so that, “[n]otwithstanding the existing scientific uncertainty”, the scientific risk assessment enable them “to ascertain, based on the best available scientific data [35] and the most recent results of international research, whether matters have gone beyond the level of risk that it deems acceptable for society” [36,37]. Exposure to a potential hazard, whether current or future, without adequate scientific evidence of the effects on human health or the environment due to intrinsic hazardous properties of a substance, does not amount to “unacceptable risk” under Article 68 REACH [38]. “Mere conjecture” is not a legally sound basis for a preventive measure, and the assertion that “all PFASs and/or their degradation products” are persistent [19] may well be mere conjecture. As remarked in Case T-13/99: “a preventive measure cannot properly be based on a purely hypothetical approach to the risk, founded on mere conjecture which has not been scientifically verified” and “a ‘zero risk’ does not exist, since it is not possible to prove scientifically that there is no current or future risk associated with” the regulated activities/products [36].

Because there is no information on the vast majority of PFASs included in the group of substances that would be subjected to proposed restrictions, the question is whether assumed persistence and projected combined adverse effects in the future are sufficient. In fact, there is at least some scientific data that contradict this hypothesis [39,40]. We deem the argument that a broad ban would “avoid regrettable substitution by other PFASs” [19] as weak, since it is not permissible to ban substances on the ground that they could be substitutes for substances that are legitimately banned. Rather than providing scientific evidence on a case-by-case basis for each of the substances subject to the proposed restriction, the proponents paint with a broad brush and merely impute hazard and risk to most of the covered substances. Persistence without “more”, which is not a hazard under the EU chemical legislation [41], is simply not enough to reach the regulatory thresholds [42,43]. At the very least, a PBT or vPvB classification of each regulated substance would be necessary before a risk caused by a substance’s persistence could rise to the level of an unacceptable risk. Further note that the standards for authorization and derogations from restriction are fundamentally different: authorizations, under the adequate control route, can be granted if risks are not unacceptable, but the basis for derogations under restrictions can only be socio-economic, since unacceptable risk would already have been established. Thus, a derogation is not equal to an authorization. Overall, the proposed PFAS ban therefore fails to meet the requirements of the REACH Regulation.

The proposed PFAS restriction has gone through a six-month public consultation, which generated more than 5600 comments, and is currently being evaluated by the ECHA’s Risk Assessment Committee (RAC). Simultaneously, the national authorities of Denmark, Germany, the Netherlands, Norway, and Sweden, who prepared the proposal, are updating their initial report to reflect the comments received. When they are finished, their report will be assessed by the RAC and SEAC. Subsequently, ECHA will submit the RAC and SEAC opinions, along with the proposed restriction, to the Commission for review and adoption through a member state committee procedure (articles 70–73, REACH Regulation).

## 6. Some Concluding Remarks

PFAS will remain a bone of (eco)toxicological contention for the foreseeable future. That fact alone lays bare the numerous mistaken perceptions of the nature of chemicals. A few of these misconceptions we touched upon in this review are as follows: “toxic freeness” is a regulatory term devoid of any real-world meaning; persistency is a physico-chemical and not a toxicological term. Considering persistency within a toxicological framework is essential to understand the (eco)toxicological concerns so far identified. That being said, persistency varies widely depending on microorganisms, degradation conditions (aerobic/anaerobic), and reaction times. Persistency, then, muddies the blanket restriction of all PFASs.

The as-yet-narrow scientific database, which is still discussed vigorously, is ostensibly strengthened by the social value test, that is, the idea of market essentiality of a given chemical. However, this takes us from the frying pan into the fire. As is well-known, many developments within the chemical field proved their (societal) usefulness over long periods of time, rendering any essentiality test moot [44]. In fact, any essentiality test would result in abandoning any and all chemical developments as such and would require de facto omniscience. Any forewarning of whatever quality and/or source that might interfere with the so-called regulatory toxic freeness of the environment can be construed as a warrant for regulation, here to be understood as a ban, as the PFAS proposal makes clear.

Finally, and to be absolutely clear, any chemical might justifiably be banned from the socio-industrial sphere if there are scientific warrants to do so. That is, however, a more complicated task than it might seem. We pointed out the chemical-analytical difficulties with reliably detecting PFAS at very low concentrations (ng/mL or L), excluding reporting measurement uncertainties, which are rarely executed. This makes for a problematic standardization of any toxicity thresholds. We also pointed out the problematic epidemiological studies we could only touch on briefly. Perhaps stating the obvious, epidemiological evidence on PFAS hazards to human health is methodologically weak yet is rarely, if ever, discussed in detail. The referred EFSA report [12] ignores all the well-known epidemiological caveats [45].

These and other scientific discussions on the topics at hand, subsequently, need to be freely accessible to any and all scientists, regulators, media, and so on. Opinions, that is, a state of affairs not known via conclusive arguments, unassailable evidence, and so on, but at best considered based on debatable arguments and not personal preferences [46], might widely diverge. And that is a good thing. Granted, this will complicate policymaking that aims at ‘certain outcomes’. That is also a good thing. The “we-have-to-do-something” fallacy rarely delivers solid outcomes.

## Data Availability

No new data were created or analyzed in this study. Data sharing is not applicable to this article.
